# Comparison of the effect of hemodialysis and peritoneal dialysis in the treatment of end-stage renal disease

**DOI:** 10.12669/pjms.39.6.8056

**Published:** 2023

**Authors:** Yuan Zhao

**Affiliations:** Yuan Zhao Department of Nephrology, The First People’s Hospital of Tonglu County, Tonglu, Hangzhou City 311500, Zhejiang Province, P.R. China

**Keywords:** Blood pressure, End-stage renal disease, Hemodialysis, Peritoneal dialysis

## Abstract

**Objective:**

To compare the clinical effects of hemodialysis (HD) and peritoneal dialysis (PD) in the treatment of end-stage renal disease (ESRD) patients.

**Methods:**

Clinical data of ESRD patients who received HD (n=74) and PD (n=77) for more than 12 months in the First People’s Hospital of Tonglu County from October 2020 to November 2021 were retrospectively selected. Renal function indexes, blood pressure, and complication rates in the two groups before the first dialysis and at the end of the observation period were compared.

**Results:**

After the dialysis, the urea nitrogen (BUN) levels decreased in both groups, and were significantly lower in the PD group compared to the HD group. Urea clearance index (Kt/V) increased, and were significantly higher in the PD group compared to the HD group (*P*<0.05). After the dialysis, albumin (ALB) and cardiac ejection fraction (EF) levels significantly increased, and ALB levels were significantly higher in the HD group while EF levels were significantly higher in the PD group (*P*<0.05). Levels of whole parathyroid hormone (iPTH), systolic blood pressure, and diastolic blood pressure indicators in both groups decreased compared to before the dialysis, and were significantly lower in the PD group compared to the HD group of patients (*P*<0.05). PD was associated with significantly lower total incidence of complications compared to HD (*P*<0.05).

**Conclusions:**

Peritoneal dialysis is more effective in maintaining the hemodynamic stability for ESRD patients, reducing blood pressure level, improving the clearance rate of molecular substances, and protecting the renal function of patients compared to hemodialysis.

## INTRODUCTION

End Stage Renal Disease (ESRD) is the most advanced stage of chronic kidney disease (CKD).^1^ With the recent socioeconomic development and lifestyle changes, ESRD has shown a rapid growth trend worldwide and has become a public health issue of global concern.^1^,^2^ The estimated incidence of ESRD is currently 373.4 per million per year, and is associated with a heavy economic burden to patients and the healthcare system.^1^-^3^

The main causes of ESRD include renal damage caused by glomerulonephritis, diabetes nephropathy, hypertensive arteriole sclerosis and polycystic kidney.^4^,^5^ ESRD patients are presenting with almost complete and irreversible renal function loss, 5 which leads to accumulation of metabolic waste and toxins, and its adverse effects, such as anemia, and uremia that is accompanied by nausea, vomiting, poor appetite, skin itching, ammonia odor, and edema.^5^,^6^

Patients with ESRD need to undergo dialysis treatment to continue and maintain their lives.^5^-^7^ Hemodialysis (HD) and peritoneal dialysis (PD) are the most commonly used methods in treating patients with ESRD.^8^ These methods are associated with various complications that may affect both the treatment and the prognosis. Therefore, it is crucial to choose the appropriate dialysis method.^7^,^9^elderly and patients with a history of cardiovascular disease. Peritoneal dialysis (PD Studies have investigated the clinical effects of HD versus PD in the treatment of ESRD patients, but the results of some indicators are inconsistent.^10^-^12^ Thus, the goal of this study was to compare renal function, changes in blood pressure and complication rates of ESRD patients treated with HD and PD.

## METHODS

A total of 151 ESRD patients (85 males and 66 females) who received dialysis treatment for more than 12 months in the First People’s Hospital of Tonglu County from October 2020 to November 2021 were selected retrospectively. The average age of the patients was 57.28 ± 8.47 years. Patients were divided into the HD group (n=74) and the PD group (n=77) according to the dialysis mode.

### Inclusion criteria:

- Patients aged > 18 years
- Conforming to ESRD clinical diagnostic criteria.^13^
- The condition is relatively stable.
- Patients without active inflammation.
- Complete clinical data.

### Exclusion criteria:

- Patients with malignant tumors.
- Severely infection.
- Patients with cerebral hemorrhage and myocardial infarction.
- Serious dysfunction of important organs (heart, liver, etc.).
- Cognitive disorders and mental diseases.
- Recent history of surgery.

### Ethical Approval

This study conforms to the relevant rules and regulations of the Ethics Committee of our hospital (Approval No.: IRB-2020-007; Date: December 30, 2020).

### Treatment

Before the HD and PD treatment, patients in both groups were given routine treatment, including prevention of infection, treatment of diabetes, heart failure, metabolic acidosis, water-electrolyte disorders, and hypovolemia. Close monitoring of patients’ basic physiological indices such as blood glucose, blood lipids, blood pressure, and heart rate, was done.

### Hemodialysis

Jinbao AK100 dialyzer and Jinbao P-14L dialyzer (Sweden) were used for the treatment. HD patients underwent percutaneous central venous intubation with bicarbonate dialysate. The surface area was 1.4 m^2^, the blood flow rate was set at 200~400 ml/minute, bicarbonate was used as the dialysate. The dialysis frequency was 2~3 times per week, and the duration of each dialysis was about 4 hours. If the patient’s blood sugar rose, subcutaneous or intravenous insulin was given to control blood sugar, and the dosage was adjusted appropriately according to the patient’s blood sugar level.

### Peritoneal dialysis

The Baite peritoneal dialysis tube and surgical incision method were used to enter the abdominal cavity, and the glucose dialysate with the concentration of 1.5%~4.5% was selected for the treatment. The flow rate was set at 200 ml/time, the frequency was four times per day, about 2000 mL per time, 4~6 hours in the daytime, 8~10 hours in the evening, and then adjusted appropriately according to the peritoneal balance test results. If the patient’s blood sugar rose, insulin was added to the peritoneal dialysis fluid, and the dosage was adjusted appropriately according to the patient’s blood sugar level. According to the patient’s condition, they can fall asleep or keep a small amount (≤ 500ml) overnight after the last dialysis fluid drainage to ensure the effectiveness of daytime dialysis.

Both groups of patients were treated with diuretics, calcium antagonists and β receptor blockers based on their blood pressure to control the blood pressure levels. The BUN, Kt/V, ALB, and iPTH, blood pressure, and complications of two groups of patients before the first dialysis and 12 months after full dialysis were recorded. The detection of serum BUN and ALB was performed using a fully automated biochemical analyzer (Hitachi, Japan). The detection of serum iPTH was carried out using chemiluminescence method (Beckman Company, USA, reagent purchased from R&D Company, USA). Kt/V formula: Kt/V=-ln (R-0.008t) + (4-3.5R) × UF/Wt, where ln is the natural logarithm; R is blood urea nitrogen after dialysis/blood urea nitrogen before dialysis; T is the time of one dialysis, unit: h; UF is the ultrafiltration amount, in liters; Wt is the patient’s weight after dialysis, in kilograms.

### Statistical Analysis

Sample size was calculated by referring to the Kt/V values of previous literature,^14^,^15^ and setting the power at 0.8, the significance level at 95%, and the possibility of incomplete records at 10%, the minimum sample size for this study was 36. The normality of the data was tested using Shapiro-Wilk test. The data of normal distribution were expressed by mean ± standard deviation. The inter group comparison was conducted by independent sample *t* test, and the intra group comparison was conducted by paired *t* test. Data of non-normal distribution were expressed by median and interquartile interval. Mann-Whitney *U* test was used for intergroup comparison, and Wilcoxon signed rank test was used for intra group comparison. Counting data were represented by the number of cases. *P*<0 .05 was the significance threshold. SPSS v26.0 (SPSS Inc., Chicago, IL, USA) was used for statistical analysis.

## RESULTS

A total of 151 patients were included in this study. The HD group included 40 males and 34 females, with the average age of 56.88±8.72 years. There were 45 males and 32 females in the PD group, with an average age of 56.67±8.27 years. There was no significant difference in baseline data between the two groups (*P*>0.05) (Table-I).

**Table-I:** Comparison of baseline information of two groups of patients.

Group	HD-group (n=74)	PD-group (n=77)	χ^2^/t/Z	p
Male (n, %)	40 (54.05)	45 (58.44)	0.295	0.586
Age (years)	56.88±8.72	56.67±8.27	-0.576	0.565
BMI (kg/m²)	24.28±2.17	23.91±2.55	0.981	0.328
***Primary diseases (n,%)***				
Glomerulonephritis	26 (35.13)	30 (38.96)	2.867	0.413
Lupus nephritis	14 (18.92)	20 (25.97)		
Renal vascular disease	19 (25.68)	12 (15.59)		
Others	15 (20.27)	15 (19.48)		
Course of ESRD (years)	8 (5, 11)	9(6, 12)	0.981	0.084
Dialysis age (months)	32 (24,39)	28 (25,35)	-1.383	0.167
Heart failure (yes)	27 (36.49)	38 (49.35)	2.547	0.110

Before the dialysis, there was no significant difference in the results of BUN and Kt/V between the two groups (*P*>0.05). After the dialysis, BUN levels in both groups decreased significantly, and were significantly lower in the PD group compared to the HD group. The Kt/V increased after the treatment, and were significantly higher in the PD group compared to the HD group (*P*<0.05) (Table-II).

**Table-II:** Comparison of BUN and Kt/V indicators between the two groups.

Group	BUN (mmol/L)		Kt/V	

	Before dialysis	After dialysis	Before dialysis	After dialysis
HD-group(n=74)	24.5 (20.6, 26.1)	19.35 (15.3, 20.9)*	1.34 (1.03, 1.64)	1.73 (1.41, 2.06)*
PD-group(n=77)	25.6 (21.3, 7.6)	16.1 (13.4, 18.7)*	1.4 (1.09, 1.69)	2.11 (1.80, 2.40)*
*Z*	-1.662	-3.287	-1.083	-4.450
*P*	0.096	<0.001	0.279	<0.001

***Note:***

* refers to the comparison between this group and before dialysis, P<0.05.

Before the dialysis, levels of ALB, iPTH, systolic blood pressure, diastolic blood pressure, and EF indicators were comparable in both groups (*P*>0.05). After the dialysis, ALB and EF levels significantly increased, and ALB levels were significantly higher in the HD group while EF levels were significantly higher in the PD group (P<0.05). Levels of iPTH, systolic blood pressure, and diastolic blood pressure indicators in both groups decreased compared to before the dialysis, and were significantly lower in the PD group compared to the HD group of patients (*P*<0.05) (Table-III). There was no significant difference in the total incidence of complications between the two groups after the dialysis (*P*<0.05) (Table-IV).

**Table-III:** Comparison of ALB, iPTH, systolic blood pressure, diastolic blood pressure, and EF index levels between the two groups.

Index	Time	HD-group (n=74)	PD-group (n=77)	Z	P
ALB (g/L)	Before dialysis	26.85(23.9, 33.1)	28.6(23.9, 34.2)	-0.867	0.386
	After dialysis	38.35(35.5, 40.0)*	36.1(34.3, 38.8)*	-2.448	<0.05
iPTH (pg/mL)	Before dialysis	633.5(587, 695)	625(565, 685)	-1.536	0.566
	After dialysis	284(254, 334)*	197(174, 250)*	-8.438	<0.001
Systolic blood pressure (mmHg)	Before dialysis	164(147, 184)	165(148, 185)	-0.507	0.612
	After dialysis	157(140, 177)*	148(124, 167)*	-2.473	0.013
Diastolic blood pressure (mmHg)	Before dialysis	86.5(84, 96)	86(78, 96)	-0.574	0.566
	After dialysis	82(79, 92)*	75(67, 85)*	-3.500	<0.001
EF(%)	Before dialysis	58(54, 62)	57(51, 62)	-0.912	0.362
	After dialysis	62(58, 66)*	65(59, 69)*	-2.155	<0.05

***Note:***

* refers to the comparison between this group and before dialysis, P<0.05.

**Table-IV:** Comparison of complication between the two groups.

Group	Complication				

	Infected	Cardiovascular disease	Hypoproteinemia	Hypoglycemia	Total
HD-group (n=74)	2(2.70)	6(8.10)	23(31.08)	2(2.70)	33(44.59)
PD-group (n=77)	1(1.29)	1(1.29)	25(32.47)	0(0.00)	27(35.06)
*χ^2^*					1.431
*P*					0.232

## DISCUSSION

This study showed that PD is associated with significant improvement in the indicators, such as BUN, KT/V, blood pressure levels, and incidence of complications compared to HD. Our results indicate that PD can control blood pressure, protect renal function, and achieve more significant clinical effects in ESRD patients more effectively than HD. Xu XD et al^16^ compared clinical effects of HD (n=44) and PD (n=40) in treating ESRD patients, and showed that PD had less impact on the blood pressure of the patients and better preserved their residual renal function, which was consistent with the results of this study. In addition, a meta-analysis study containing 12 randomized controlled trials (n=932) by Zou M et al.^17^ showed that PD was associated with lower incidence of cardiovascular and cerebrovascular events and bleeding complications in ESRD patients. However, although the incidence of complication in the PD group in this study was lower than that of the HD group, there was no significant difference, which may be attributed to the small sample size and selection bias of this study.

Studies shows that HD can easily lead to the increase of blood pressure in patients, while PD has better control effect on blood pressure.^16^,^17^ We speculated that various factors such as the different degrees of arteriosclerosis in ESRD patients, or hemodynamic changes during the course of the treatment, may lead to an overall high rate of cardiovascular diseases such as hypertension.^18^ ESRD hypertension is not only associated with increased pressure in the glomerulus, but also with the process of glomerulus aging and hardening, which eventually increases the degree of renal failure and overall mortality in long-term dialysis patients.^19^ During dialysis, excessive and fast dehydration may lead to a sudden drop in blood volume, and subsequent decrease in the renal artery perfusion pressure. Additionally, it will activate the renin-angiotensin system, and lead to an increase in the level of angiotensin-II, upregulation of endothelin secretion, contraction of renal vessels, and elevation of blood pressure. This, in turn, will lead to the instability of blood flow mechanics, thus forming a vicious circle.^20^ HD is characterized by intermittent dehydration. During the treatment, the patient’s water intake is excessive. After the dialysis and accompanying dehydration, the patient’s blood pressure continues to rise due to excessive dehydration, and the hemodynamics is unstable, which may further aggravate the disease.^21^ In addition, HD patients may have bleeding complications such as puncture site bleeding, residual blood in dialyzer and pipeline, and easy coagulation during cardiopulmonary bypass.^21^,^22^ PD, on the other hand, uses patient’s own peritoneum as a dialysis membrane, so this treatment is the closest to the physiological state, which can ensure the relative stability of the renin-angiotensin system.^23^ PD can simulate the continuous dialysis of kidney, the dehydration process is slow and continuous. It can continuously and slowly remove toxins and excessive water load, reduce the range of blood volume changes, ensure the stability of hemodynamics, and improve the stability of its internal environment. PD, therefore, not only reduces the incidence of cardiovascular and other complications, but also effectively controls blood pressure and protects the residual renal function of patients.^23^,^24^ PD can also achieve continuous replacement of dialysate through peritoneal diffusion and ultrafiltration, which can effectively remove metabolic wastes and uremic toxins from the body and protect its renal function.^25^ Additionally, PD requires only establishing arteriovenous fistula, which can ensure the balance of acid-base and degree-electrolyte in the body, eliminate the accumulation of toxins in the body, protect renal function, and further improve the level of urea nitrogen and creatinine.^23^,^25^

During maintenance dialysis, dietary restrictions and simultaneous filtration of small molecular nutrients (amino acids, Vitamin B12) may worsen the nutritional status of the patients, leading to hypoproteinemia and anemia.^26^,^27^ Meanwhile, due to the decrease in appetite caused by dialysis, it may also lead to a decrease in the patient’s food intake.^26^ Therefore, nutritional issues during dialysis are important factors that affect the overall treatment effectiveness of patients.^27^ Studies have shown that PD is superior to HD in improving malnutrition.^28^,^29^ However, the results of our study showed that after the dialysis, ALB levels in both groups of patients were significantly higher than before the dialysis, but still lower than normal levels. Moreover, iPTH levels in both group after treatment were significantly lower than those before the dialysis. These indexes in the PD group was significantly lower than in the HD group, indicating that PD was not as effective in improving malnutrition of ESRD patients as HD. The reason may be due to the bias in the sample size of this study. In addition, it may be related to insufficient protein energy intake of patients, abdominal fullness caused by peritoneal dialysis fluid, absorption of high glucose in peritoneal dialysis fluid or PD-related peritonitis.^30^ As malnutrition can lead to an increased risk of infection, hospitalization, and death, PD patients should be given a high protein diet appropriately to improve their quality of life.^28^-^30^

### Limitation of the study

It is a single-center retrospective study with small sample size and short observation time, which may lead to some bias in the judgment of efficacy. Further follow-up studies with larger sample size that will observe the survival rate of patients, and further explore the dialysis scheme of ESRD patients, are needed.

## CONCLUSION

Compared with HD, PD treatment can effectively maintain the hemodynamic stability of ESRD patients, reduce blood pressure level, improve the clearance rate of molecular substances, effectively protect the renal function of patients.

### Authors’ Contributions:

**YZ:** Conceived and designed the study.

**YZ:** Collected the data and performed the analysis.

**YZ:** Was involved in the writing of the manuscript and is responsible for the integrity of the study.

All authors have read and approved the final manuscript.
